# In vitro evaluation of flow patterns and turbulent kinetic energy in trans-catheter aortic valve prostheses

**DOI:** 10.1186/1532-429X-17-S1-Q33

**Published:** 2015-02-03

**Authors:** Daniel Giese, Bettina Baessler, Navid Madershahian, Yeong-Hoon Choi, David Maintz, Alexander Bunck

**Affiliations:** 1 University Hospital Cologne, Cologne, Germany

## Background

Trans-catheter aortic valve implantation (TAVI) has emerged as an alternative to open heart surgery for valve replacement. Hemodynamics in these bioprostheses however still differ from physiological conditions. Multi-venc phase-contrast MRI is known to allow the quantification of flow parameters and recent developments enable the quantification of turbulent kinetic energy (TKE). Turbulent flows do not generally occur under physiological conditions and are known to lead to an increased workload for the heart. In the present work, highly accelerated multi-venc phase-contrast MRI is used to compare flow patterns and TKE values in different TAVI prostheses in a pulsatile in-vitro model.

## Methods

Three different TAVI prostheses were measured using highly accelerated 3D phase-contrast MRI in a 3T Achieva (Philips Medical) system using a 6-channel cardiac coil. Physiological pulsatile flow waveforms were achieved using a conventional ventricular assist device (VAD). The prostheses were positioned in a flexible PVC tube (diameter: 19 mm). Blood-like viscosity was achieved by using a glycerol (40%) and water (60%) mixture. Multi-venc acquisition parameters included venc: 350&70&40cm/s, Δx=1.5mm^3^, Δt=40ms, undersampling factor:8. Measurements were reconstructed using *k-t* Principal Component Analysis (*k-t* PCA) and unfolded using a Bayesian approach allowing for concurrent flow and TKE calculations. Analysis was performed using GTFlow (Gyrotools).

## Results

3D velocity streamlines showed drastically different flow patterns, especially distal to the different TAVI prostheses in peak systole (Figure). TKE values differed in location, spatial extent and also in maximum values ranging from 150 to over 300 J/m^3^. For the 3 valves, the quantification of the mean TKE within a 3D volume 7cm distal to the valve resulted in 30.1, 69.65 and 68.35 J/m3. The maximum TKE value within a cross-sectional slice was 63.4, 94.5 and 111.68 J/m3 and was reached at levels 10.5mm, 36mm and 18mm distal to the valve.

**Figure 1:**
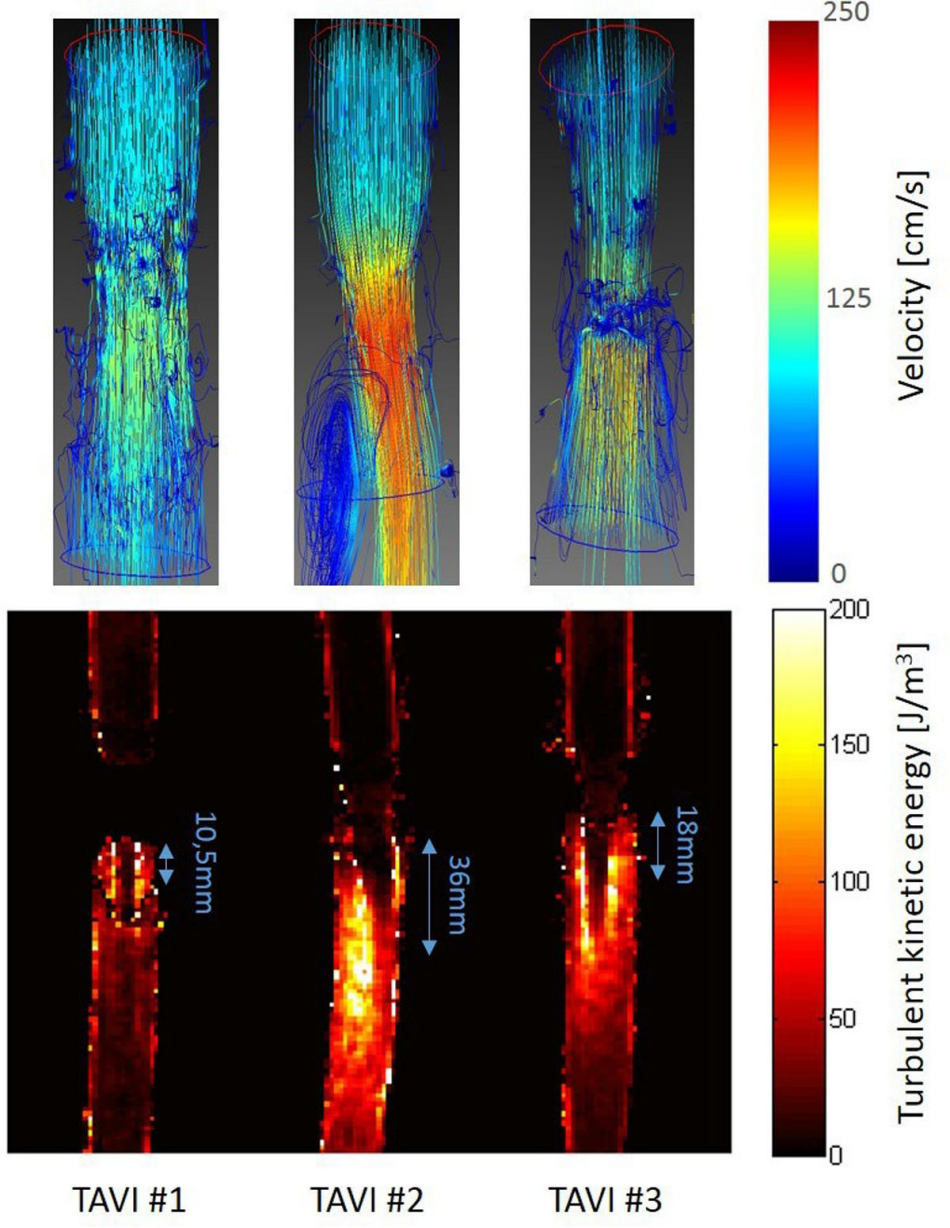
Color-coded path lines (top) and TKE maps (bottom) in early systole of the 3 TAVI prostheses (flow direction: top to bottom).

## Conclusions

Highly undersampled multi-venc 3D phase-contrast MRI allows for the evaluation of time-resolved 3D flow parameters, flow patterns as well as the quantification of turbulent kinetic energy. The first acquisition of TKE values in TAVI prostheses demonstrated different flow patterns and TKE values for 3 different prostheses. The acquisition time of under 10 minutes allows future work to include in vivo acquisitions of healthy subjects and patients prior and following TAVI implantation.

## Funding

Supported by the Koeln Fortune Program / Faculty of Medicine, University of Cologne.

